# Association of Interleukin-6 Genetic Polymorphisms (rs1800795, -174C > G and rs1800796, -572G > C) With Risk of Essential Hypertension in the Chinese Population

**DOI:** 10.7759/cureus.46334

**Published:** 2023-10-01

**Authors:** Huan Ren, Zhen Guo, Wen-jie Qin, Zhi-ling Yang

**Affiliations:** 1 Pharmacy, Hunan Provincial People's Hospital, The First Affiliated Hospital of Hunan Normal University, Changsha, CHN; 2 Genetics, Hunan Provincial Key Laboratory of the Fundamental and Clinical Research on Functional Nucleic Acid, Changsha Medical University, Changsha, CHN

**Keywords:** inflammatory factor, single nucleotide polymorphism, interleukin-6, essential hypertension, genetic polymorphism

## Abstract

Background

Interleukin-6 (*IL-6*) plays a critical role in essential hypertension (EH) and cardiovascular disease. Evidence suggests two hotspot single nucleotide polymorphisms (SNPs) of the *IL-6* gene (rs1800795, -174C > G and rs1800796, -572G > C) might be associated with the susceptibility of EH. However, no consensus has yet been established. Thus, we aimed to investigate the potential association between *IL-6* gene polymorphisms and the risk of EH based on a case-control study in a Chinese population.

Materials and methods

A total of 479 subjects (272 healthy controls and 207 EH patients) were randomly enrolled in our study. After extracting the genomic DNA, two SNPs of the *IL-6* gene (rs1800795, -174C > G and rs1800796, -572G > C) were genotyped to analyze the potential association between these genetic variants and EH risk. Multiple genetic models were performed to investigate the strength of association by calculating the odds ratio (OR) and 95% confidence interval (95% CI). The potential effect of SNPs on gene expression was evaluated using expression quantitative trait loci (eQTL) analysis.

Results

The genotyping findings of *IL-6* rs1800795, -174C > G polymorphism showed three study participants with CG genotype and 204 with GG genotype in the EH patients. The *IL-6* -174C > G polymorphism was significantly associated with EH risk (*P* = 0.046) and conferred a reduced risk of EH development (OR = 0.99, 95%CI = 0.97-1.00). Conversely, no substantial association between *IL-6* rs1800796, -572G > C polymorphism and the risk of EH was found in all genetic models (*P* > 0.05). Moreover, the eQTL analysis indicated that the -174C > G polymorphism was significantly associated with gene expression of *IL-6* (*P* = 0.006), and the G allele corresponded to a reduced *IL-6* gene expression (Beta = -0.397). Compared with -174C > G, the -572G > C polymorphism was not found to be significantly associated with *IL-6* gene expression (Beta = -0.120, *P* = 0.560).

Conclusions

Our findings provide evidence that the rs1800795, -174C > G polymorphism can affect the expression levels of *IL-6*, and the risk of EH occurrence. However, the rs1800796, -572G > C polymorphism does not regulate the *IL-6 *gene expression levels and the susceptibility of EH.

## Introduction

Hypertension is a leading risk factor for cardiovascular events and a significant contributor to death and disability globally [1]. Owing to population aging, unhealthy lifestyles, and lack of physical activity, the prevalence of hypertension is rising especially in developing countries [2]. The priority should be given to fully understanding the molecular mechanisms of hypertension and to control this trend.

Over the past few years, while many factors have been shown to lead to the pathogenesis of hypertension, a crucial role of the immune system in the development of hypertension has been consistently established by accumulating information on vascular inflammation [3]. Interleukin-6 (*IL-6*), produced by macrophages, T cells, and endothelial cells, is an important multifunctional cytokine that plays a pivotal role in the regulation of the immune system [4]. The inhibition of individual IL-6 ameliorates the development of experimental hypertension. Studies in the genetically modified mouse (*IL-6*^−/−^ mice) strains have demonstrated that deletion of *IL-6 *can prevent AngII-induced hypertension [5]. When it comes to clinical studies, less consistent results were found in the association between *IL-6* levels and the risk of hypertension [6,7].

Single nucleotide polymorphisms (SNPs) are the most common genetic variations for genomic association studies. The functional SNPs in the promoter region may regulate the transcriptional expression [8-10]. Two hotspot SNPs have been detected in the promoter of the human *IL-6* gene, that is, the C to G transition in the 174 base pairs (bp) and the G to C transition in the 572 bp upstream from the start site of transcription. The two promoter polymorphisms (rs1800795, -174C > G and rs1800796, -572G > C) may be associated with alterations in the transcription activity of *IL-6*, although the geographical and ethnic distribution of these polymorphisms are quite different [11,12]. The genetic variants located in the regulatory region are more likely to regulate the activity of gene expression and affect the risk of diseases. Therefore, we sought to examine the association of the two promoter polymorphisms inthe* IL-6 *gene with essential hypertension (EH) risk among the Chinese population and assess their effects on the *IL-6 *gene expression levels.

## Materials and methods

Study participants

The participants included in this study were 479 unrelated Chinese Han individuals, who were recruited from a department of cardiology and health management center. Patients with EH were diagnosed according to the universal diagnostic criteria, a resting systolic blood pressure (SBP) above 140 mmHg and/or diastolic blood pressure (DBP) above 90 mmHg, or taking antihypertensive drugs. The subjects with secondary hypertension, such as renal artery stenosis, pyelonephritis, adrenal adenoma, coarctation glomerulonephritis, and hyperaldosteronism, were excluded. The participants in the control group were selected from individuals who attended a routine checkup from the same demographic but had no history of hypertension. The exclusion criteria for the controls were hypertension, coronary heart disease, and diabetes. The clinical baseline information, including age, sex, blood pressure, alcohol and smoking habits, were collected. The study protocol was conducted in accordance with the Declaration of Helsinki guidelines and approved by the Human Research Ethics Committee. Informed consent for inclusion was acquired from the participants in the study.

Sample collection and genotyping of *IL-6* SNPs

Around 3-5 mL of peripheral blood from the patients with EH and controls were collected in vials containing ethylenediaminetetraacetic acid. The genomic DNA was extracted from the whole blood samples using a DNA extraction kit (OMEGA) based on the manufacturer's protocol. The concentration, purity, and integrity of the extracted DNA samples were determined by the nano-drop method and agarose gel electrophoresis. All extracted genomic DNA was stored at −80 °C until required for further analysis. The genotyping of *IL-6* SNPs was performed using MassARRAY matrix-assisted laser desorption/ionization time-of-flight mass spectrometry.

Expression quantitative trait loci (eQTL) analysis

To explore the potential effects of -174C > G and -572G > C polymorphisms on *IL-6* gene expression, we used the NephQTL project database in our study [13]. The MatrixEQTL was applied to perform the eQTL analysis by adjusting for several critical covariates, including patient age, sex, and microarray batch.

Statistical analysis

All the data analysis was performed using the IBM SPSS statistics software version 20.0 (IBM, USA). The frequencies of genotype and allele were compared by direct counting. The linkage disequilibrium (LD) analysis was evaluated using the online software SHEsis (http://analysis.bio-x.cn/myAnalysis.php). Deviations from Hardy-Weinberg equilibrium (HWE) were performed by a chi-square test. The association of the two* IL-6* genetic polymorphisms (-174C > G and -572G > C) with the risk of EH was evaluated using the odds ratios (ORs) and 95% confidence intervals (CIs). A *P *value of < 0.05 was considered a statistically significant difference.

## Results

Basic information of subjects and selected SNPs in *IL-6*

**Figure 1:**
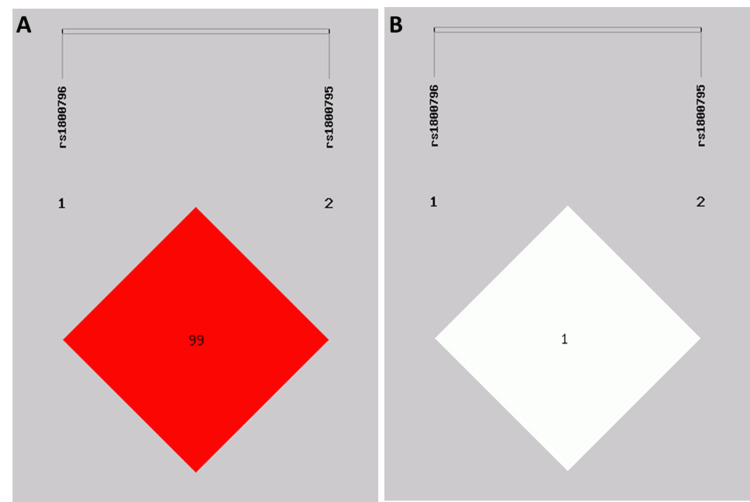
The IL-6 rs1800796 (-572G > C) and rs1800795 (-174C > G) polymorphisms are independent of each other. (A) D’-value (×100) and (B) r2-value (×100) of linkage disequilibrium analysis for the two polymorphisms

There were 479 subjects in this study, namely, 272 healthy controls and 207 EH patients. Table 1 shows the basic characteristics of the selected SNPs in *IL-6*. The two SNPs, rs1800796 (-572G > C) and rs1800795 (-174C > G), were located in the 5'-untranslated region (5'-UTR) of *IL-6*. The genotyping results showed that the alternative allele frequencies of -572G > C and -174C > G predominated in our study population, similar to the data from East Asia of the 1000G Phase 3 population. In addition, the linkage disequilibrium (LD) analysis of the two SNPs was calculated on the basis of case and control samples. As depicted in Figure 1, *IL-6* -572G > C and -174C > G are independent of each other (r^2^ = 0.012, D’ = 0.994).

**Table 1:** The basic information and linkage disequilibrium tests of selected SNPs in IL-6. EAS: East Asia of 1000G Phase 3 population; LD: Linkage disequilibrium; Ref > Alt: Reference allele > Alternative allele; SNPs: Single nucleotide polymorphisms

Gene	SNPs	Chromosome: Position	Ref > Alt	Function annotation	Alternative allele frequencies		LD calculation	
					EAS population	Our population	r²	D'
IL-6	rs1800796	7:22726627	G > C	5'-UTR	79.1%	78.8%	0.012	0.994
	rs1800795	7:22727026	C > G	5'-UTR	99.9%	99.7%		

Association of *IL-6* rs1800796 (-572G > C) polymorphism with the risk of EH development

From Table 2, it is shown that the genotype distribution of the rs1800796 (-572G > C) polymorphism between the control group and patients with EH was in HWE (controls, X^2^ = 3.045, *P* = 0.081 vs. patients with EH, X^2^ = 1.292, *P* = 0.256). The CC genotype was found most frequently, followed by the GC and GG genotypes, both in the patients with EH and controls.

**Table 2:** Observed and expected genotypes of IL-6 -174C > G and -572G > C polymorphisms in EH cases and controls. EH: Essential hypertension; SNPs: Single nucleotide polymorphisms

SNPs	Genotypes	Control				EH			
		Observed	Expected	X^2^ value	P-value	Observed	Expected	X^2^ value	P-value
rs1800796, -572G > C	GG	7	11.74	3.045	0.081	7	9.78	1.292	0.256
	GC	99	89.53			76	70.43		
	CC	166	170.74			124	126.78		
rs1800795, -174C > G	CC	0	0	0.000	1.000	0	0.01	0.011	0.916
	CG	0	0			3	2.98		
	GG	272	272			204	204.01		

To determine whether the *IL-6* -572G > C polymorphism was associated with the risk of EH occurrence, five different genetic models were performed, that is, allelic genetic model (C-allele vs. G-allele), heterozygote genetic model (GC vs. GG), homozygote genetic model (CC vs. GG), recessive genetic model (CC vs. GG+GC), and dominant genetic model (CC+GC vs. GG). No substantial difference was found in the distribution of *IL-6* -572G > C genotype, as well as G and C alleles between controls and patients with EH. Thus, no notable association between IL-6 -572G > C polymorphism and EH risk was also found in all genetic models, such as the heterozygote (OR = 0.77, 95%CI = 0.26-2.28, *P* = 0.634), homozygote (OR = 0.75, 95%CI = 0.26-2.19, *P* = 0.593), dominant (OR = 0.76, 95%CI = 0.26-2.19, *P* = 0.603), recessive (OR = 0.95, 95%CI = 0.66-1.38, *P *= 0.803), and allelic (OR = 0.94, 95%CI = 0.69-1.29, *P* = 0.717) inheritance (Table 3).

**Table 3:** Genetic association of IL-6 -174C > G and -572G > C polymorphisms with risk of hypertension. EH: Essential hypertension; NA: Not available; OR: Odds ratio; 95% CI: 95% confidence interval. SNPs: Single nucleotide polymorphism; *P < 0.05 in bold was considered statistical significance

SNPs	Models	Genotypes	Control	EH	OR (95% CI)	P-value
rs1800796, -572G > C	Reference	GG	7 (2.6%)	7 (3.4%)	1	
	Heterozygote	GC	99 (36.4%)	76 (36.7%)	0.77 (0.26-2.28)	0.634
	Homozygote	CC	166 (61.0%)	124 (59.9%)	0.75 (0.26-2.19)	0.593
	Reference	G	113 (20.8%)	90 (21.7%)	1	
	Allelic	C	431 (79.2%)	324 (78.3%)	0.94 (0.69-1.29)	0.717
	Reference	GG	7 (2.6%)	7 (3.4%)	1	
	Dominant	CC+GC	265 (97.4%)	200 (96.6%)	0.76 (0.26-2.19)	0.603
	Reference	GG+GC	106 (39.0%)	83 (40.1%)	1	
	Recessive	CC	166 (61.0%)	124 (59.9%)	0.95 (0.66-1.38)	0.803
rs1800795, -174C > G	Reference	CC	0 (0.0%)	0 (0.0%)	1	
	Heterozygote	CG	0 (0.0%)	3 (1.4%)	NA	NA
	Homozygote	GG	272 (100.0%)	204 (98.6%)	NA	NA
	Reference	C	0 (0.0%)	3 (0.7%)	1	
	Allelic	G	544 (100%)	411 (99.3%)	0.99 (0.99-1.00)	0.047
	Reference	CC	0 (0.0%)	0 (0.0%)	1	
	Dominant	GG+CG	272 (100.0%)	207 (100.0%)	NA	NA
	Reference	CC+CG	0 (0.0%)	3 (1.4%)	1	
	Recessive	GG	272 (100%)	204 (98.6%)	0.99 (0.97-1.00)	0.046

Association of *IL-6* rs1800795 (-174C > G) polymorphism with the risk of EH development

For the rs1800795 (-174C > G) polymorphism, the genotype distribution in the research samples was also in HWE (X^2 ^= 0.011, *P* = 0.916). It was identified that the *IL‑6* ‑174 GG genotype was the most prevalent, while the CG genotype was less common, and the CC genotype was not observed in patients with EH. However, both CG and CC genotypes were not found in the control group (Table 2).

As shown in Table 3, different genetic models were performed to investigate the strength of association between -174C > G polymorphism and susceptibility of EH, that is, allelic genetic model (G-allele vs. C-allele), heterozygote genetic model (CG vs. CC), homozygote genetic model (GG vs. CC), recessive genetic model (GG+CG vs. CC), and dominant genetic model (GG vs. CC+CG). The results indicated that substantial association between -174C > G polymorphism and EH risk in the allelic (*P* = 0.047) and recessive (*P* = 0.046) genetic model of inheritance. Therefore, compared to the C allele carriers, the G allele (OR = 0.99, 95%CI = 0.99-1.00) and GG genotype (OR = 0.99, 95%CI = 0.97-1.00) appear to be a protective factor for EH development.

Effect of rs1800796 (-572G > C) and rs1800795 (-174C > G) polymorphisms on the gene expression of *IL-6*

**Figure 2:**
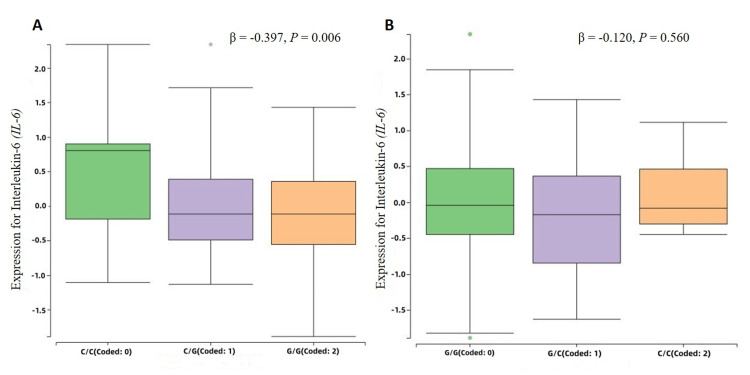
Effects of IL-6 rs1800796 (-572G > C) and rs1800795 (-174C > G) polymorphisms on the gene expression. (A) The -174C > G polymorphism contributes to a reduced gene expression of IL-6. (B) The -572G > C polymorphism do not regulate IL-6 gene expression. β > 0 and β < 0 indicate that the alternative allele regulates increased and reduced gene expression, respectively. P-value < 0.05 denotes statistical significance.

An eQTL analysis was used to evaluate the association of *IL-6* genetic polymorphisms with gene expression levels. Using the NephQTL database, the rs1800795 (-174C > G) was found to be significantly associated with differential gene expression of IL-6 (*P* = 0.006), and the G allele corresponded to a substantial decrease of *IL-6* gene expression (Beta = -0.397), as described in Figure 2A. However, in contrast with -174C > G polymorphism, rs1800796 (-572G > C) was not significantly associated with *IL-6* gene expression (Beta = -0.120, *P* = 0.560), as described in Figure 2B.

## Discussion

Recently, much attention has been given to the roles of inflammatory factors or the immune system in the pathogenesis of hypertension [14]. In this study, we investigated whether associations existed between the two promoter hotspot SNPs (rs1800795, -174C > G and rs1800796, -572G > C) in the *IL-6* gene and the susceptibility of hypertension in the Chinese population. Our findings provide evidence that the *IL-6* -174C > G polymorphism can affect its gene expression levels and risk of hypertension. However, the -572G > C variant does not lead to the change of *IL-6 *gene expression and the development of hypertension.

IL-6, a multifunctional cytokine, plays a key role in the development of cardiovascular diseases, such as hypertension, atherosclerosis, and kidney diseases [15]. In fact, some genetic association studies also have investigated the role of *IL-6* polymorphisms in the development of hypertension individually, but the previous findings are inconsistent [16-18]. Jeng et al. found a positive association between the* IL-6* −174GG genotype and risk of hypertension and elevated plasma plasminogen activator inhibitor-1 level in the Taiwan Chinese population [16]. However, the polymorphism was not found to be associated with hypertension incidence in a prospective cohort of white population [17]. In the Hong Kong Chinese population, plasma *IL-6* can be affected by the variant rs1800796, while this SNP was not associated with blood pressure or hypertension [18].

Very few studies have investigated the two promoter hotspot polymorphisms at the same time in the same case-control population till now. In the present study, our results showed that frequencies of genotypes bearing the C allele of *IL-6* rs1800795 (-174C > G) were only found in patients with hypertension enabling us to conjecture that the presence of the G allele might be protective against the progression to hypertension. As for the *IL-6* gene rs1800796 (-572G > C) polymorphism, no substantial associations were found in all genetic models, indicating the mutation did not contribute to the occurrence of hypertension. Additionally, the previously reported frequency of the two polymorphisms varied across varying research based on different genotyping methods [16,18]. High-accuracy genotyping methods were the basis of quality control. Consistent with the frequency in eastern Asian populations from 1,000 genomics data, the -174C > G polymorphism also had a high mutation allele frequency in our population. Likewise, the frequency of -572G > C polymorphism was in good agreement with data from 1,000 genomics in eastern Asian populations. These data indicated the high accuracy of the two polymorphisms genotyping results in our study and ensured the objectivity and precision of our conclusion.

The molecular mechanism of how *IL-6* genetic variants affect the risk of EH occurrence remains to be clarified. It has been assumed that functional variations in the promoter region of the *IL-6* gene may influence the transcriptional activity by its responsiveness to inflammatory stimuli-mediated activation [19]. The eQTL analysis is a key method to access the functional roles of non-coding genetic variations [10]. In our study, we investigated the relationship of genetic variations with *IL-6* gene expression via the NephQTL dataset. The eQTL results can account for the different association of *IL-6* -174C > G and -572G > C with hypertension risk, as the -174G allele leads to the lower expression of *IL-6*, while the -572G > C polymorphism does not affect the expression of *IL-6* in eQTL analysis. Similar to our findings, a previous clinical study also indicated that the promoter hypomethylation of *the IL-6* gene may enhance essential hypertension risk by upregulating the expression levels of IL-6 [20]. Therefore, the regulation of gene expression could be the mechanism via which the *IL-6* genetic polymorphisms influence the occurrence of EH.

## Conclusions

In conclusion, the present study indicated that the rs1800795, -174C > G polymorphism in the promoter region contributed to a lower expression of *IL-6* and was significantly associated with a reduced risk of EH susceptibility, while no notable association of rs1800796, -572G > C polymorphism with the expression of *IL-6*, and the risk of EH occurrence was found in the Chinese population. Our findings showed that the effect of *IL-6* genetic variants on the essential hypertension phenotype was small. Unraveling the genetic associations could potentially help us better understand the mechanisms or underlying biological pathways of essential hypertension.
